# *In Vivo* Detection of Amyloid-β Deposits Using Heavy Chain Antibody Fragments in a Transgenic Mouse Model for Alzheimer's Disease

**DOI:** 10.1371/journal.pone.0038284

**Published:** 2012-06-04

**Authors:** Rob J. A. Nabuurs, Kim S. Rutgers, Mick M. Welling, Athanasios Metaxas, Maaike E. de Backer, Maarten Rotman, Brian J. Bacskai, Mark A. van Buchem, Silvère M. van der Maarel, Louise van der Weerd

**Affiliations:** 1 Department of Radiology, Leiden University Medical Center, Leiden, The Netherlands; 2 Department of Human Genetics, Leiden University Medical Center, Leiden, The Netherlands; 3 Nuclear Medicine and PET Research, Radionuclide Center, Free University Medical Center, Amsterdam, The Netherlands; 4 Department of Neurology, Massachusetts General Hospital and Harvard Medical School, Charlestown, Massachusetts, United States of America; 5 Department of Anatomy and Embryology, Leiden University Medical Center, Leiden, The Netherlands; Northwestern University Feinberg School of Medicine, United States of America

## Abstract

This study investigated the *in vivo* properties of two heavy chain antibody fragments (V_H_H), ni3A and pa2H, to differentially detect vascular or parenchymal amyloid-β deposits characteristic for Alzheimer's disease and cerebral amyloid angiopathy. Blood clearance and biodistribution including brain uptake were assessed by bolus injection of radiolabeled V_H_H in APP/PS1 mice or wildtype littermates. In addition, *in vivo* specificity for Aβ was examined in more detail with fluorescently labeled V_H_H by circumventing the blood-brain barrier via direct application or intracarotid co-injection with mannitol. All V_H_H showed rapid renal clearance (10–20 min). Twenty-four hours post-injection ^99m^Tc-pa2H resulted in a small yet significant higher cerebral uptake in the APP/PS1 animals. No difference in brain uptake were observed for ^99m^Tc-ni3A or DTPA(^111^In)-pa2H, which lacked additional peptide tags to investigate further clinical applicability. *In vivo* specificity for Aβ was confirmed for both fluorescently labeled V_H_H, where pa2H remained readily detectable for 24 hours or more after injection. Furthermore, both V_H_H showed affinity for parenchymal and vascular deposits, this in contrast to human tissue, where ni3A specifically targeted only vascular Aβ. Despite a brain uptake that is as yet too low for *in vivo* imaging, this study provides evidence that V_H_H detect Aβ deposits *in vivo*, with high selectivity and favorable *in vivo* characteristics, making them promising tools for further development as diagnostic agents for the distinctive detection of different Aβ deposits.

## Introduction

Besides neurofibrillary tangles, Alzheimer's disease (AD) is characterized by cerebral deposition of β-amyloid (Aβ) in so-called senile or diffuse plaques [1]. Similar vascular deposits of Aβ associated with cerebral amyloid angiopathy (CAA) lead to loss of vessel wall integrity increasing the risk of brain haemorrhages [2]. Present in 30% of the non-demented population over 60 years of age, CAA co-exists in 90% of the AD patients and forms an important complication in the development of immunotherapeutic strategies [3]–[5]. Although, the exact role of Aβ regarding the underlying pathogeneses remains unsolved, accumulation is believed to start 20–30 years prior to clinical onset [6], [7]. Distinctive *in vivo* detection of the different Aβ deposits therefore renders important knowledge regarding early diagnosis and preventive therapy development.

Currently, a gross differentiation can only be made based on the occipital predilection of CAA, while existing PET ligands, like ^11^C-PiB, target Aβ in its fibrillar amyloid form rather than specific vascular or parenchymal types of Aβ deposits [8].

Previously, we have selected heavy chain antibody fragments with high affinity specific for either CAA or all types of human Aβ deposits [9]. Derived from the Camelid heavy chain antibody repertoire, which completely lack light chains, their single N-terminal domain (V_H_H) is fully capable of antigen binding with affinities comparable with those of conventional antibodies [10], [11].

Blood-brain barrier (BBB) passage was shown to be favorable in an *in vitro* assay [12]; therefore, this study assessed the *in vivo* characteristics of two distinct Aβ targeting V_H_H, ni3A and pa2H, for their potential use to differentially detect AD and CAA. First, pharmacologic behaviour and biodistribution were examined after administration of radiolabeled V_H_H into a transgenic AD/CAA mouse model. Secondly, fluorescently labeled V_H_H were administered after the BBB was circumvented to evaluate their ability to specifically bind Aβ deposits *in vivo*.

**Figure 1:**
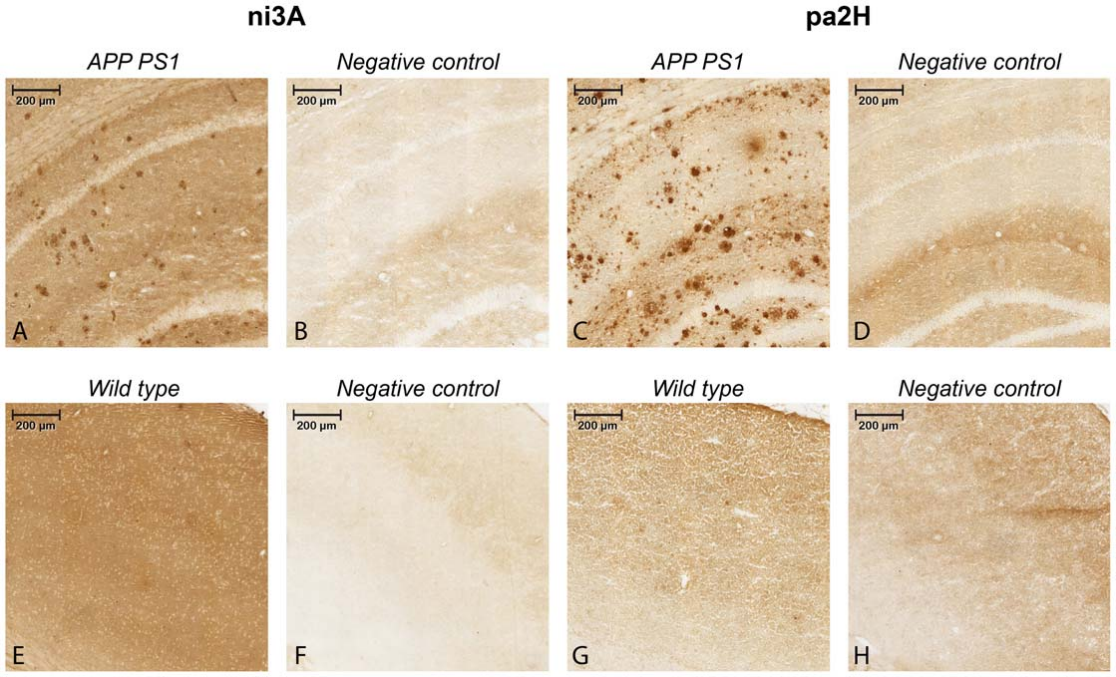
Immunostaining on murine APP/PS1 sections using ni3A and pa2H. The upper panels (**A–D**) show 10× magnifications of the resulting staining with cryosections of aged APP/PS1 mouse brain tissue including negative controls, while the lower panels (**E–H**) show similar staining performed with wildtype littermates.

## Materials and Methods

### Production of ni3A and pa2H

V_H_H ni3A and pa2H were selected from respectively a non-immune or an immune library created after immunisation with post-mortem brain parenchyma of a patient with Down's syndrome. V_H_H were subcloned and produced as previously reported including a myc- or VSV-tag for detection and a *his*-tag for purification [9]. Similarly, pa2H free of any additional peptide tags was commercially produced by overexpression in yeast (BAC, Leiden, the Netherlands).

### Animal studies

All studies were performed using 12–16 month old transgenic mice or wildtype littermates from a colony set up using the APPswe/PS1dE9 strain (APP/PS1) (JAX), known to accumulate vascular and parenchymal Aβ deposits [13], and have been approved by the institutional Animal Ethics Committee (DEC) at the Leiden University Medical Center, permit number 09132. Besides standard genotyping, after each experiment amyloid pathology was confirmed by standard Thioflavin T staining.

**Table 1:** Biodistribution of ^99m^Tc-ni3A in mice.

	t = 3 hr		t = 6 hr		t = 24 hr	
Tissue/organ	Wildtypes	APP/PS1	Wildtypes	APP/PS1	Wildtypes	APP/PS1
blood	1.202*±0.379*	1.146*±0.131*	0.778*±0.048*	0.808*±0.115*	0.451*±0.073*	0.363*±0.051*
heart	0.525*±0.129*	0.508*±0.109*	0.347*±0.049*	0.337*±0.127*	0.252*±0.041*	0.216*±0.014*
lungs	0.850*±0.184*	0.819*±0.208*	0.659*±0.184*	0.743*±0.301*	0.375*±0.113*	0.291*±0.055*
liver	1.078*±0.235*	1.000*±0.293*	1.078*±0.188*	1.223*±0.424*	0.568*±0.149*	0.488*±0.164*
kidneys	15.531*±2.986*	15.192*±3.075*	10.266*±1.657*	14.294*±4.337*	9.089*±6.152*	9.901*±1.158*
spleen	0.590*±0.257*	0.531*±0.084*	0.792*±0.144*	0.753*±0.291*	0.397*±0.056*	0.465*±0.234*
muscle	0.171*±0.075*	0.120*±0.069*	0.111*±0.088*	0.086*±0.022*	0.043*±0.007*	0.048*±0.008*
cerebrum	0.035*±0.009*	0.035*±0.007*	0.031*±0.007*	0.035*±0.008*	0.018*±0.003*	0.019*±0.001*
cerebellum	0.073*±0.031*	0.063*±0.009*	0.098*±0.009*	0.096*±0.014*	0.029*±0.005*	0.026*±0.002*
cerebrum/blood ratio	0.030*±0.003*	0.030*±0.004*	0.040*±0.010*	0.043*±0.004*	0.040*±0.001*	0.053*±0.008* *
cerebrum/muscle ratio	0.242*±0.142*	0.335*±0.116*	0.407*±0.270*	0.428*±0.171*	0.422*±0.029*	0.403*±0.100*

* *= P<0.05 wildtype mice compared to APP/PS1 mice.*

A bolus injection of 2 µg ^99m^Tc-ni3A was administered intravenously into 12–14 month old APP/PS1 mice or their wild type littermates. At three time points after injection the animals were sacrificed and various tissues and entire organs were removed, weighed and counted for radioactivity. Values are expressed as a percentage of the injected dose per gram tissue (mean ± SD).

### Human material

Human brain tissue was obtained of AD/CAA patients or controls as confirmed by neuropathological examination in agreement with the guidelines of the ethics committee of the LUMC. Patient anonymity was strictly maintained. All tissue samples were handled in a coded fashion, according to Dutch national ethical guidelines (Code for Proper Secondary Use of Human Tissue, Dutch Federation of Medical Scientific Societies).

**Table 2:** Biodistribution of radiolabeled pa2H in mice.

	^99m^Tc-pa2H						DTPA(^111^In)-pa2H	
	t = 3 hr		t = 6 hr		t = 24 hr		t = 24 hr	
Tissue/organ	Wildtypes	APP/PS1	Wildtypes	APP/PS1	Wildtypes	APP/PS1	Wildtypes	APP/PS1
blood	0.566*±0.003*	0.654*±0.015*	1.009*±0.054*	1.244*±0.123*	0.575*±0.084*	0.696*±0.049*	0.006*±0.001*	0.004*±0.002*
heart	0.273*±0.121*	0.240*±0.017*	0.623*±0.101*	0.763*±0.031*	0.367*±0.059*	0.393*±0.007*	0.017*±0.084*	0.014*±0.003*
lungs	0.843*±0.256*	0.537*±0.010*	0.930*±0.242*	1.088*±0.035*	0.620*±0.160*	0.622*±0.031*	0.016*±0.011*	0.014*±0.003*
liver	2.615*±0.796*	1.866*±0.016*	3.014*±1.021*	3.392*±1.932*	1.430*±0.402*	1.161*±0.470*	0.066*±0.029*	0.075*±0.0.23*
kidneys	9.243*±1.787*	6.241*±0.530*	14.306*±4.105*	15.612*±1.042*	9.824*±2.810*	8.608*±0.738*	8.859*±3.623*	7.689*±2.930*
spleen	1.515*±0.503*	1.319*±0.060*	6.498*±1.623*	6.258*±0.208*	3.584*±1.381*	1.747*±0.100*	0.044*±0.022*	0.048*±0.006*
muscle	0.356*±0.379*	0.054*±0.006*	0.174*±0.022*	0.347*±0.026*	0.102*±0.023*	0.113*±0.018*	0.059*±0.020*	0.059*±0.044*
cerebrum	0.014*±0.003*	0.017*±0.001*	0.033*±0.005*	0.044*±0.004*	0.027*±0.004*	0.038*±0.002* *	0.001*±0.000*	0.001*±0.001*
cerebellum	0.023*±0.001*	0.026*±0.001*	0.054*±0.016*	0.067*±0.001*	0.030*±0.007*	0.045*±0.000* *	0.003*±0.001*	0.002*±0.001*
cerebrum/blood ratio	0.025*±0.005*	0.026*±0.003*	0.033*±0.004*	0.035*±0.004*	0.047*±0.003*	0.055*±0.008*	0.013*±0.011*	0.041*±0.055*
cerebrum/muscle ratio	0.083*±0.081*	0.309*±0.067*	0.190*±0.007*	0.177*±0.135*	0.270*±0.032*	0.346*±0.377*	0.113*±0.066*	0.203*±0.146*

* *= P<0.05 wildtype mice compared to APP/PS1 mice.*

A bolus injection of 2 µg radiolabeled pa2H was administered intravenously into 12–14 month old APP/PS1 mice or their wildtype littermates. At three or one time points after injection of radiolabeled pa2H respectively with or without additional peptide tags, the animals were sacrificed and various tissues and entire organs were removed, weighed and counted for radioactivity. Values are expressed as a percentage of the injected dose per gram tissue (mean ± SD).

### Murine specificity of the selected V_H_H

To evaluate appropriate use of the APP/PS1 mouse model, murine cryosections (10 µm) were stained according previous protocols [9], [12] with in addition a standard anti-mouse-to-mouse kit (ARK, Dako Cytomation). Final preparations were analyzed with an automated Pannoramic MIDI microscope (3DHistech).

### Biodistribution and clearance

#### Radiolabeling

V_H_H were labeled according to two different protocols. First, *his*-tagged V_H_H were labeled directly with technetium-99m (^99m^Tc) using a previously published protocol [14]. Briefly, 20 µl of V_H_H in PBS solution (450–500 ng/µl) was added to 8 µl of an aseptic mixture of 950 mg/l Sn(Cl)_2_.2H_2_O and 2 g/l Na_4_P_2_O_7_.10H_2_O (Technescan PYP, Covidien, Petten, the Netherlands) in saline. After addition of 4 µl of 10 mg/ml of KBH_4_ (crystalline, Sigma Chemical Co, St. Louis, MO) in 0.1 M NaOH, and 100 µl of Na[^99m^TcO_4_] solution (approximately 200–700 MBq/ml, Technekow, Covidien, Petten, the Netherlands) the mixture was gently stirred at room temperature for at least 30 min before use. Analysis of the labeling solution, referred to as ^99m^Tc-V_H_H, yielded a radiochemical purity of >95% without detectable unreduced or free ^99m^TcO_4_ [15].

Secondly, untagged V_H_H were chelated for indium-111 (^111^In) using diethylene triamine penta-acetic acid (DTPA). Untagged pa2H was chelated in a total volume of 1.0 ml with 20-fold molecular excess of *p*-SCN-Bn-DTPA (Macrocyclics, Dallas, TX) at pH 8.5 in phosphate buffer for 5 hr at 37.5°C and purified by dialysis using phosphate buffered saline (PBS). ^111^In chloride (25 µl, 111 MBq/ml, Covidien, the Netherlands) was added to DTPA-pa2H conjugate (0.1 ml) in 0.25 M ammonium acetate buffer (0.8 ml) at pH 5.5 and incubated for 1 hr at room temperature. The reaction was quenched with 50 mM ethylene diamine tetra-acetic acid (EDTA) (50 µl) to chelate residual non-bound ^111^In and the radiolabeled antibody was then purified using a Sephadex™ G-25 column (PD 10; GE Healthcare) eluted with PBS. Radiochemical purity assessed by instant thin layer chromatography (ITLC) yielded a purity of >95%.

#### Biodistribution and brain uptake

To study the biodistribution, animals were injected intravenously with 0.2 ml radiolabeled V_H_H diluted with saline (5–10 MBq/ml, 10 µg/ml). At different intervals (t = 3–6–24 hrs) post-injection APP/PS1 (n = 4) and wildtype animals (n = 4) were sacrificed (Euthanasol, AST Pharma). Similar biodistribution experiments using untagged DTPA(^111^In)-pa2H were only performed at 24 hours post-injection for APP/PS1 (n = 6) and wildtype mice (n = 6). Blood was collected via cardiac puncture, and various organs were removed, including the brain, which was divided into the cerebrum and cerebellum. All were weighed and counted for radioactivity (Wizard^2^, Perkin Elmer). After decay correction, radioactivity was expressed as the percentage of the total injected dose of radioactivity per gram tissue (%ID/g). Blood/cerebrum ratios were calculated to correct for possible confounding effects accountable by residual blood. Similarly, muscle/cerebrum determined target-to-non-target ratios. Differences were regarded significant when *p*≤0.05 using an unpaired one or two tailed *t*-test. Experiments at t = 24 hrs were repeated twice using ^99m^Tc-pa2H.

**Figure 2:**
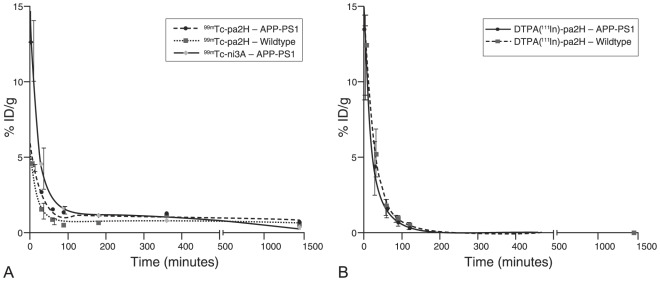
Blood clearance. These graphs represent the blood half lives of tagged ^99m^Tc-ni3A and -pa2H (**A**), and untagged DTPA(^111^In)-pa2H (**B**) in APP/PS1 mice and wildtype littermates. Data is shown as percentage of injected dose per gram of blood (%ID/g) over time. Based upon this plot the clearance is suggested to respectively consist of a fast and a slow phase, or only a single phase.

**Table 3:** Blood half lives of radiolabeled V_H_H.

		Fast *t_½_*			Slow *t_½_*		
V_H_H	genotype	*(min)*	*(95% C.I.)*	%	*(min)*	%	*(95% C.I.)*
^99m^Tc-ni3A	APP/PS1	14.71	*(8.65–49.13)*	89.7	580	10.3	*(101.8–∞)*
	Wildtype	*ND*		*ND*	*ND*	*ND*	
^99m^Tc-pa2H	APP/PS1	21.89	*(14.24–39.38)*	79.8	2562	20.2	*(975.0–∞)*
	Wildtype	10.78	*(7.27–20.76)*	87.1	5861	12.9	*(969.3–∞)*
DTPA(^111^In)-pa2H	APP/PS1	19.69	*12.63–44.60*	100	-	-	
	Wildtype	15.83	*9.30–53.37*	100	-	-	

Half lives were determined by fitting a one or a two phase exponential decay model based on blood obtained from both tail vein and cardiac puncture at several time points after intravenous bolus injection of 2 µg radiolabeled V_H_H in 12–14 month old APP/PS1 mice and wildtype littermates, as depicted in Figure 2. Please note that DTPA(^111^In)-pa2H was produced without any additional peptide tags.

**Table 4:** Blood distribution of ^99m^Tc-pa2H.

Sample	Time p.i.	Fraction	APP/PS1		Wildtype	
	*(min)*		*(%)*	*sd*	*(%)*	*sd*
^99m^Tc-pa2H	10	Plasma	88,9	6,2	80,3	5,2
		Cell Pellet	11,1		19,7	
	90	Plasma	83,6	8,7	72,0	8,2
		Cell Pellet	16,4		28,0	

At different time point after bolus injection of ^99m^Tc-pa2H blood collected from the tail vein of 12–14 month old APP/PS1 mice or wildtype littermates. Separated into the cell pellet and plasma, samples were counted for radioactivity. Fractions are expressed in percentage of total activity at that time point. No significant differences were calculated using a student *t*-test (*p*<0.05).

#### Blood clearance and analysis

Simultaneously, blood half-life was examined by collecting 5 µl tail samples at several time points between 3–90 minutes post-injection of radiolabeled V_H_H into transgenic or wildtype mice (n = 4–6). Combined with the cardiac blood samples corresponding half-lives were calculated using GraphPad Prism.

Similarly, 10 µl samples obtained at 10 and 90 minutes post-injection were mixed with 90 µl heparin (34 U/ml saline) and 900 µl PBS. Centrifugation for 10 minutes at 7,000 rpm separated plasma from the cell pellet. Radioactivity was measured separately to determine the blood distribution of radiolabeled V_H_H over time.

#### Specificity of radiolabeled pa2H

Aβ specificity of pa2H-*his* after ^99m^Tc-radiolabeling was tested by quantitative competition autoradiography. Human and murine brain cryosections (20 µm) were blocked with 1% bovine serum albumin (BSA)/PBS at 37°C for 1 hour followed by similar application of the labeling solution, which was diluted to 1 µg/ml by 1%BSA/PBS with or without additional 1 hour pre-incubation with excess monomeric or fibrillar Aβ_1–40_ (rPeptide) at 37°C. Fibrils were produced using existing protocols [16].

After rinsing 3 times with PBS, radioactivity was counted for 15 minutes by a gamma camera (Toshiba GCA7100/UI). A similar region of interest was fitted for each scintigram to assess binding of ^99m^Tc-pa2H-*his* with 0.1 ml of diluted labeling solution as a reference. Binding was expressed as the % of radioactivity compared to the section without any competitor. Experiments were performed in triplicate.

**Table 5:** Quantitative autoradiography.

Brain tissue	Binding of ^99m ^Tc-V_H_H	Competion binding	
		Monomeric Aβ	Fibrillar Aβ
	ng (± sd)	ng (± sd)	ng (± sd)
APP/PS1	98.8 (±20,7)*	60.1 (±22.2)	31.4 (±14.3)
Wildtype	86.4 (±14.8)	56.4 (±19.5)	27.1 (±12.5)
AD human	190.1 (±73.5)*	81.4 (± N.D.)	42.3 (±29.2)
Control human	102.3 (±30.2)	27.2 (± N.D.)	49.9 (±17.4)

Differences in radioactivity were measured after application of 1 µg ^99m^Tc-pa2H to human and murine APP/PS1 brain sections.

* Statistical difference (*p*<0.05) between either murine or human control versus Aβ bearing sections.

### *In vivo* Aβ targeting by V_H_H

#### Fluorescent labeling

Tagged V_H_H were fluorescently labeled with Alexa Fluor 594 protein labeling kit (Molecular Probes, Invitrogen) according to the manufacturer's guidelines, except using only half of the recommended amount of dye. Briefly spun to remove possible aggregates, extensive dialysis removed excess free label. The labeling degree and protein concentration (200–600 ng/µl) were determined using the Nanodrop ND1000 (Isogen Life Sciences). Protein integrity was confirmed by mass spectrometry.

#### Immunofluorescence using VHH-Alexa594

To examine whether the fluorescent labeling affected antigen recognition, human and murine cryosections (10 µm) were rinsed with PBS, fixed in ice-cold acetone for 10 minutes before overnight incubation with V_H_H- Alexa594 in 1% BSA/PBS in a wet chamber. Washed 3×5 minutes with PBS, sections were mounted and analyzed using a fluorescence microscope (Leica DMR5500B).

#### In vivo Aβ imaging by topical application

Four APP/PS1 animals received permanent cranial windows to allow serial *in vivo* imaging of the brain by multiphoton microscopy. Animals were anaesthetized using 2% isoflurane gas inhalation, and the exposed skull was partly replaced by a round glass coverslip glued into place using Krazyglue® according to previous surgical protocols [17], [18]. Prior to fixation of the cranial window, a drop of 40–60 µl of V_H_H-Alexa594 (275–400 ng/µl) was applied directly onto the exposed brain for 30 minutes and briefly rinsed with PBS. Colocalization with the Aβ deposits was based either upon their typical green autofluorescence or by intraperitoneal injections of Methoxy-X04 one day prior surgery [16]. Animals were imaged immediately following surgery, which was typically less than 90 minutes after beginning of the procedure, and re-imaged under isoflurane anaethesia (2%) for several days to study the washout. Images were acquired with a Bio-Rad 1024 multiphoton microscope equipped with a Ti:Sapphire laser (Mai Tai, Spectra Physics) and external photodetectors (Hamamatsu Photonics). Areas were imaged to approximately 200 µm deep in 5 µm steps with a 20× objective (UMPlanFl, NA = 0.95; Olympus). Maximum intensity projections were reconstructed using ImageJ.

#### Specific in vivo Aβ binding after BBB disruption

A systemic approach to study the in vivo behaviour of the VHH throughout a larger area within the brain involved intracarotid infusion (60 µl/min) of 100 µl pa2H-his-Alexa594 along with 600 µl 15% mannitol selectively into the right carotic artery to disrupt the BBB [19]. At t = 2 and 24 hours post-injection., transgenic (n = 9) and wildtype animals (n = 3) were euthanized (Euthanasol, AST Pharma), and perfused with 4% paraformaldehyde (PFA). Resected brains were stored in 4% PFA with 10% sucrose for 4 hours followed by overnight fixation in 4% PFA with 30% sucrose. Next, the brains were snap frozen and sectioned completely to obtain consecutive 30-µm-thick cryosections. Besides standard Thioflavin T staining for amyloid, adjacent sections were immunostained for Aβ (6F/3D, DakoCytomation) [20] with 1∶100 goat-antimouse-Alexa488 (Invitrogen) to assess colocalization. Images obtained by a Leica DM5500B microscope were merged using Adobe Photoshop CS3.

## Results

### Murine specificity of the selected V_H_H

Immunostained brain sections of aged APP/PS1 and wildtype littermates using tagged V_H_H ni3A and pa2H were made to assess their capacity to selectively recognize different types of deposits. (Figure 1) Pa2H stained positive for all forms of Aβ depositions. In this transgenic mouse model, ni3A did not show selective affinity for vascular Aβ; both vascular and parenchymal Aβ depositions were clearly labeled. Compared to ni3A, equivalent staining protocols with pa2H resulted in higher specificity for Aβ combined with a low unspecific background binding. For neither V_H_H specific affinity was detected within the brain sections of wildtype animals.

**Figure 3:**
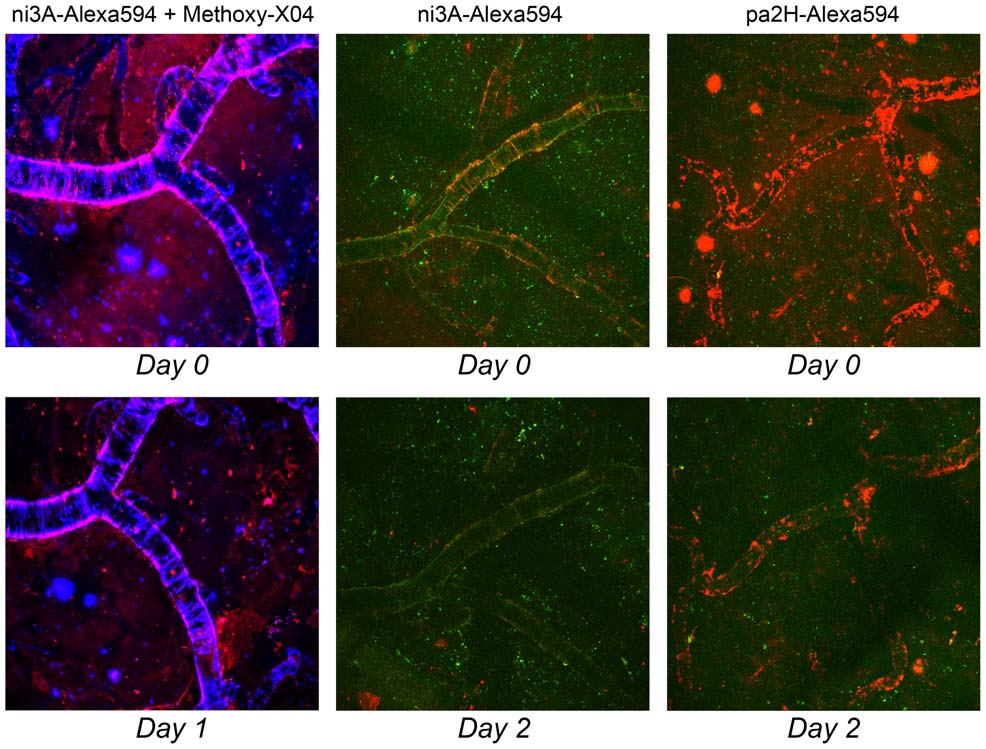
*In vivo* Aβ imaging after direct brain application. Topical application of ni3A- or pa2H-Alexa594 (*red*) as visualized over time by intravital multiphoton microscopy in APP/PS1 mice clearly shows the specific *in vivo* labeling of different Aβ deposits. In the ***left***, vascular and parenchymal Aβ deposits, detected by prior labeling with Methoxy-X04 (blue), colocalize with ni3A-Alexa594 (red) directly following topical application. One day later, labeling of the plaques has diminished to almost none with some residual left bound to CAA. With interpretation hampered by Methoxy-X04, ***middle*** images show a similar experiment. Colocalization with Aβ deposits based upon autofluorescence (*green*) gave comparable results and almost complete wash out after two days. Pa2H-Alexa594 (red), as shown in the ***right*** images, remains bound to vascular Aβ even two days after application, when the plaques remained undetected. All images are maximum intensity projections of a 3D cortical volume with a field of view 615×615 µm.

### Biodistribution and clearance

#### Biodistribution and brain uptake

The distribution of a bolus injection of radiolabeled tagged ni3A and pa2H over time is shown in Table 1 and 2. No significant differences in organ uptake between wildtype and transgenic animals were found, except for the brain uptake of ^99m^Tc-pa2H after 24 hours. Although the amount was low (0.038%I.D./g), cerebral uptake was 40% higher in the transgenic animals. The cerebrum/blood ratio did not differ, indicating that this difference was not caused by different V_H_H concentrations within the blood pool. For the cerebellum similar results were found. Repeated experiments for this particular endpoint resulted in similar findings.

**Figure 4:**
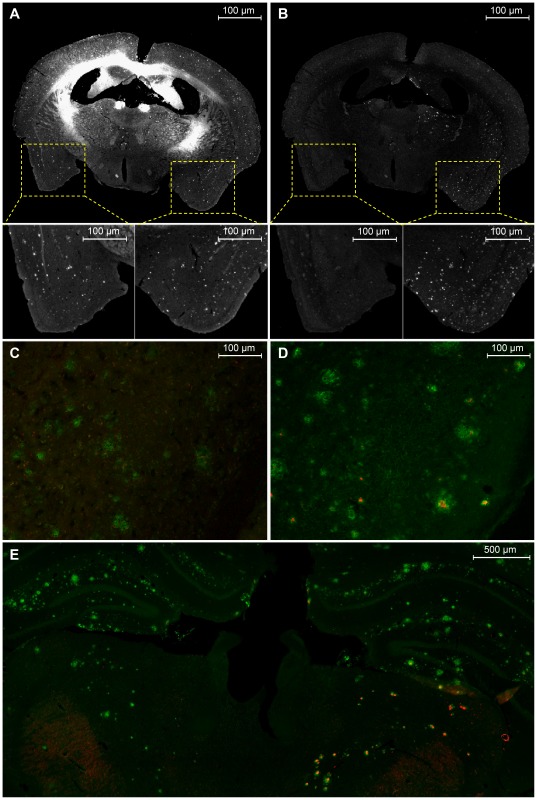
Specific *in vivo* Aβ binding after BBB disruption. After disruption of the BBB using a co-injection of 15% mannitol with pa2H-Alexa594 into the right carotid artery of an aged APP/PS1 mouse sacrificed 2 hrs post injection, amyloid plaques are clearly depicted in both hemispheres using a Thioflavin T (ThT) staining (**A**), while the pa2H-Alexa594 signal is only detected in the right hemisphere (**B**). More careful examination shows all Alexa594 signal colocalizes with ThT in the right hemisphere, while in the left only some autofluorescense can be detected. Furthermore, immunofluorescense anti-Aβ staining of the plaques using Alexa488 within the left hemisphere (**C**) results only in green signal, while within the right hemisphere (**D**) the red signal from pa2H-Alexa594 nicely colocalizes within the plaques. Experiments performed in a similar setting but sacrificed 24 hrs post-injection, showed similar results with pa2H-Alexa594 still nicely corresponding to the green labeling of the anti-Aβ staining within the right hemisphere (**E**).

**Figure 5:**
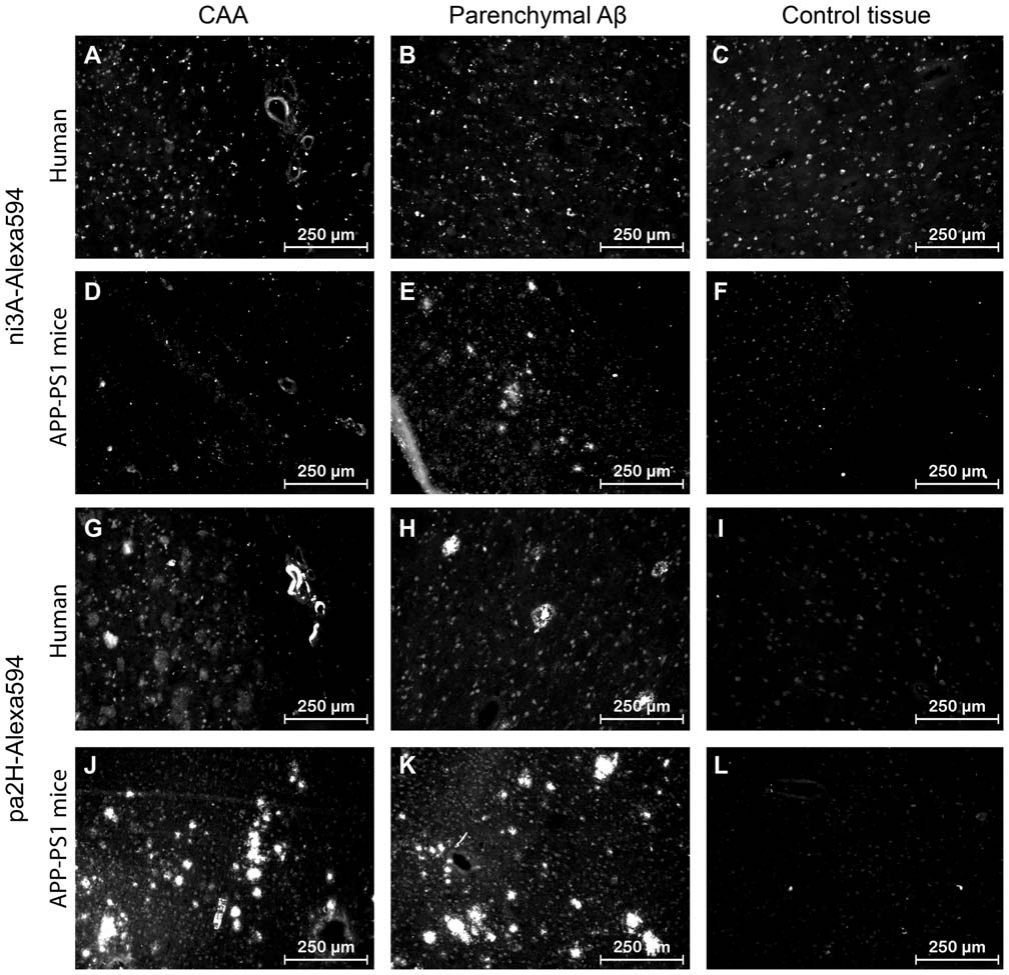
Immunofluorescence with V_H_H-Alexa594. Shown are the results of immunofluorescence staining with ni3A- and pa2H-Alexa594 on cryosections of APP/PS1 murine and human AD/CAA brain tissue, including wildtype or healthy controls. Both V_H_H stain positive for CAA in all sections (**A**, **D**, **G**, **J**). Only ni3A-Alexa594 stained negative for human parenchymal Aβ (**B**), while pa2H stained positive for several types of parenchymal Aβ deposits (**G**, **H**, **J**, **K**) in both humans and mice. In either human of murine control tissue no such staining patterns were observed.(**C**, **F**, **I**, **L**)

To investigate whether these findings were not confounded by either the non-specific radiolabeling procedure or the presence of additional peptide tags, the biodistribution experiment was repeated with untagged DTPA(^111^In)-pa2H. (Table 2) With this labeling protocol, we no longer observed a significantly higher cerebral uptake in amyloid-bearing mice. Regardless of the tag, the majority of radiolabeled V_H_H was excreted via the kidneys. Cellular involvement as shown by distinctive hepatic clearance or splenal activity was low. In comparison to ^99m^Tc-ni3A, ^99m^Tc-pa2H showed about 3 times higher clearance via liver and spleen. Also, the clearance rate for ^99m^Tc-pa2H was lower, independent of genotype. However, within the first 3 hours ^99m^Tc-ni3A resulted in a higher general organ uptake, with exception of the aforementioned liver and spleen.

#### Blood clearance and analysis

Blood clearance of the tagged ^99m^Tc-V_H_H consisted of a fast and a slow component. (Figure 2A) In general, the majority of the radiolabeled V_H_H was cleared from the blood with a half-life of 10–20 minutes (Table 3). The actual half-life of the slow component of ^99m^Tc- V_H_H could only be calculated with limited accuracy, since the half-life was longer than the blood sampling period. In line with the above biodistribution, six hours post-injection, the blood levels of ^99m^Tc-pa2H were remarkably higher compared to earlier time points, which is characteristic for a second passage. Within the first 90 minutes about 80% of the ^99m^Tc-V_H_H remained within the blood plasma, indicating that no significant cellular uptake occurred. (Table 4)

In contrast to tagged ^99m^Tc- V_H_H, the blood clearance of untagged DTPA(^111^In)-pa2H was mono-exponential, with a similar rapid clearance within 20 minutes, but without a slow component. (Figure 2B)

#### Specificity of 99mTc-pa2H

After radiolabeling of the tagged pa2H it's specificity for Aβ was unaffected, as shown by scintigraphic analysis; binding of ^99m^Tc-pa2H was higher in those sections including Aβ. (Table 5) Furthermore, binding was significantly (p<0.001) reduced when the tracer was pre-incubated with either monomeric or fibrillar Aβ.

### In vivo Aβ targeting by V_H_H

#### In vivo Aβ imaging by topical application

After direct application onto the exposed mouse brain, fluorescent V_H_H were followed up for at least 48 hours by *in vivo* multiphoton microscopy. (Figure 3) Specific *in vivo* labeling of Aβ plaques by ni3A-Alexa594 was initially confirmed by colocalization with Methoxy-X04, a known *in vivo* amyloid targeting fluorophore. Beside possible binding competition with the V_H_H, Methoxy-X04 hampered good validation due to signal cross-over into the red channel. However, colocalization based on the typical autofluorescence patterns of the different Aβ deposits resulted in similar findings. Selectivity was confirmed by lack of nonspecific background signal. Although both V_H_H were capable of targeting Aβ *in vivo*, only pa2H-Alexa594 was detectable after two days, mainly bound to vascular amyloid.

#### Specific in vivo Aβ binding after BBB disruption

Based on the above findings, co-injections of pa2H-Alexa594 with mannitol were done in the right carotid artery to selectively open the BBB in the ipsilateral hemisphere to study the in vivo characteristics throughout the brain. Two hours post-injection, fluorescence was detected in the right hemisphere, co-localizing with Aβ. (Figure 4) Even within the deeper brain structures, no nonspecific binding was observed. Aβ related fluorescent signal remained detectable for at least 24 hours post-injection. Without BBB disruption or within wildtype littermates, no apparent Aβ labeling could be detected.

#### Immunofluorescence using VHH-Alexa594

Selectivity for specific Aβ deposits was not altered after fluorescent labeling of the VHH, since on human sections, ni3A-Alexa594 selectively stained vascular Aβ (Figure 5 A–C), and pa2H-Alexa594 stained both parenchymal and vascular Aβ.(Figure 5 G–I) On murine material all Aβ deposits were stained by both fluorescent VHH.(Figure 5 D–F & J–L)

## Discussion

In this study, we assessed two previously described V_H_H for their potential to cross the blood-brain barrier and distinctively detect vascular and parenchymal Aβ deposits *in vivo*.

### Specific detection of parenchymal and vascular amyloid in APP/PS1 mice

Both V_H_H stained positive for Aβ upon APP/PS1 brain sections confirming appropriate use of this transgenic model. *In vivo* binding to parenchymal and vascular Aβ was confirmed when the BBB was circumvented. Signal remained detectable for at least 24 hours while *in vivo* pa2H showed a high affinity combined with a low off-rate. However, previously shown selectivity for solely vascular Aβ in human post-mortem brain sections by ni3A was not observed within this mouse model (Figures 1,3,5). Fluorescent or radiolabeling prior *in vivo* application did not affect their specificity. The unique specific reactivity of ni3A for vascular amyloid deposition on human brain material is not yet completely understood [9]. Known differences in morphology and composition of human and murine Aβ deposits might help to understand ni3A's specific reactivity [21], [22]. Human plaques consist of discontinuous patches with decreased density and random fibrillar orientation within the amyloid core; murine plaques are generally built up by long organized fibrils, resulting in densely packed amyloid plaques with a relatively large core [23]. Besides morphological differences, posttranslational modifications of Aβ differ from mouse to man leading to alterations of the Aβ molecule itself [21], [24], [25]. Differences in metal ion content are known to influence the tertiary structure [26], [27]. Previous epitope mapping revealed that ni3A has no other cross reaction but to Aβ_1–42_ [9], which is highly abundant in parenchymal and vascular deposits in both humans and APP/PS1 mice. All together, this leads to the conclusion that the selective reactivity of ni3A must depend on the structural presentation of Aβ_1–42_, in which case murine parenchymal plaques probably show structural similarities to human CAA.

### *In vivo* blood-brain barrier passage

Previous *in vitro* data suggested that our V_H_H actively migrated across the BBB in a more efficient way than FC5, a V_H_H specifically selected to pass the BBB [12]. However, the *in vivo* experiments resulted only in a small cerebral uptake of the tagged ^99m^Tc-pa2H at 24 hours after intravenous administration, and the current brain uptake levels were insufficient to assess the uptake kinetics *in vivo* with for example SPECT imaging. (data not shown) Additional experiments with untagged DTPA(^111^In)-pa2H further confirmed the current limitations as hardly any cerebral uptake was observed with this labeling protocol. The increased brain uptake for 99mTc-pa2H compared to DTPA(^111^In)-pa2H may be due to the slower blood clearance for ^99m^Tc-pa2H. The observed fast blood clearance and relatively high renal retention for the VHH in this study is in line with previous reports [28], [29], and typical for peptides and proteins smaller than the filtering threshold of the glomerular membrane (<60 kDa) [30]. However, in general, a short blood residential time effectively reduces the blood-to-brain transfer.

*In vivo* studies with the BBB crossing V_H_H FC5 demonstrated 4%ID/g brain uptake, which is much higher than our findings [31]. This discrepancy may be due to the lower dose that we used, but several other factors may also play a part. For FC5 it is known it uses receptor-mediated endocytosis via the α(2,3)-sialoglycoprotein [32]. For our V_H_H, *in vitro* active transport mechanisms are involved, but the specific receptors are as yet unknown [12]. Possibly, the *in vivo* BBB passage may be limited by the availability of these receptors in our mouse model.

To improve BBB penetration for the amyloid-targeting V_H_H, one could increase the blood circulation time by multimerization or by conjugating the V_H_H to an albumin-targeting moiety or V_H_H [33], [34]. An alternative approach would be to incorporate the V_H_H into a BBB-targeting nanoparticle. Recently, several nanoparticle carrier systems have been developed for brain delivery of therapeutics that would also be suitable for loading with V_H_H [35].

### Diagnostic and therapeutic value of V_H_H

In general, V_H_H constitute many unique characteristics that make them interesting tools for either diagnostics or therapeutics. Compared to conventional monoclonal antibodies or Fab', V_H_H express a similar unique level of specificity and affinity, but because of their single domain, production and modification is relatively easy and cost-efficient [29].

Currently used amyloid-targeting ligands, like ^11^C-PiB recognize amyloid plaques rather than Aβ. In contrast, we already showed that V_H_H may be more specific to a certain sub-types of Aβ accumulation [9]. Further selection may allow the *in vivo* detection of the full range of Aβ aggregates from oligomers to dense core plaques to CAA.

Besides diagnostics, several V_H_H have shown their potential therapeutic value in vitro, preventing aggregation of amyloid fibrils, oligomeric forms of Aβ and polyA-binding protein nuclear 1 [36]–[39]. In the latter case, even complete clearance of existing aggregates was reported. Whether V_H_H evaluated in this study possess similar abilities is currently under investigation. However, within the data presented here, we observed that several Aβ plaques, as detected by their autofluorescence, could no longer be seen two days after V_H_H application. (Figure 3) Whereas current passive immunotherapies targeting Aβ are hampered by unwanted immunogenic side effects, repetitive administration of V_H_H has shown to be non-immunogenic [4], [40]. Furthermore, their selective binding to different Aβ species, like ni3A's specific binding for CAA, could shift Aβ brain efflux in the favored direction, which could be used to tailor anti-Aβ therapy to further reduce therapy-induced complications, e.g. CAA related microbleeds [4], [5], [41].

These initial *in vivo* studies to investigate whether Aβ specific V_H_H can be exploited as diagnostic tools show promising results for further development. Although capable of strong specific binding *in vivo* with low unspecific background binding and favorable wash-out, issues regarding higher brain uptake and clearance need to be addressed in the future.
